# Oncolytic adenovirus encoding variant interleukin-2 combined with chemotherapy enables PD-L1 inhibition in pancreatic cancer models

**DOI:** 10.1007/s00262-025-04072-6

**Published:** 2025-06-04

**Authors:** Santeri A. Pakola, Nea Ojala, Tatiana V. Kudling, James H. A. Clubb, Elise Jirovec, Mirte van der Heijden, Victor Arias, Lyna Haybout, Saru Basnet, Susanna Grönberg-Vähä-Koskela, Dafne C. A. Quixabeira, Joao M. Santos, Victor Cervera-Carrascon, Otto Hemminki, Anna Kanerva, Harri Mustonen, Pauli Puolakkainen, Hanna Seppänen, Akseli Hemminki

**Affiliations:** 1 Cancer Gene Therapy Group, Translational Immunology Research Program, Faculty of Medicine, University of Helsinki, Helsinki, Finland; 2 TILT Biotherapeutics Ltd, Helsinki, Finland; 3 Helsinki University Hospital Comprehensive Cancer Center, Helsinki, Finland; 4 Department of Urology, Helsinki University Hospital, Helsinki, Finland; 5 Department of Gynecology and Obstetrics, Helsinki University Hospital, Helsinki, Finland; 6 Department of Surgery, Translational Cancer Medicine Research Program and ICAN Digital Precision Cancer Medicine Flagship, University of Helsinki and Helsinki University Hospital, Helsinki, Finland

**Keywords:** Oncolytic virus, Adenovirus, Pancreatic cancer, Immunotherapy, PD-L1, Checkpoint inhibitor

## Abstract

Pancreatic ductal adenocarcinoma (PDAC) is a cancer with dismal prognosis due to resistance to most current therapies. Although immunotherapy has improved the treatment of many solid cancers, pancreatic cancer remains resistant to immunotherapy due to immunosuppressive tumor microenvironment, limited lymphocyte infiltration and lack of neoantigens. Oncolytic adenoviruses are a possible solution to treatment resistance in PDAC due to their ability to elicit lymphocyte trafficking and epitope spreading. Herein, we tested if an oncolytic adenovirus encoding a variant interleukin-2 molecule (Ad5/3-E2F-d24-vIL2), could enable immune checkpoint inhibitor (ICI) therapy in PDAC when combined with chemotherapy. Rationale for Ad5/3-E2F-d24-vIL2 was tested in vitro, where increase in programmed death ligand 1 (PD-L1) expression was seen after virotherapy and chemotherapy. Expression of other B7 family proteins was characterized in mono- and co-culture settings of cancer cells, fibroblasts, and macrophages. The combination therapy of virotherapy, chemotherapy and ICI was characterized in freshly resected ex vivo pancreatic tumor samples. Combination of ICI with virotherapy showed increased interferon and chemokine production in samples, with expansion of cytotoxic CD8 + T cells seen by flow cytometry. In vivo evaluation of the triple combination therapy in a Syrian hamster model showed improved tumor growth control and overall survival, with concurrent increase in intratumoral lymphocytes during therapy. Animals cured with the therapy showed resistance to re-challenge with the same cell line, supportive of successful generation of anti-tumor immunity in the animals. The combination treatment of Ad5/3-E2F-d24-vIL2, chemotherapy, and checkpoint inhibition is a promising treatment modality to tackle treatment resistance in PDAC.

**Supplementary Information:**

The online version contains supplementary material available at 10.1007/s00262-025-04072-6.

## Introduction

Pancreatic cancer is a markedly treatment resistant cancer with extremely poor prognosis [1]. The most prevalent form of pancreatic cancer, pancreatic ductal adenocarcinoma (PDAC), is projected to become the second leading cause of cancer-related death in the USA by 2030, driven by increasing incidence rates and limited progress in treatment modalities [2, 3]. Current treatment of pancreatic cancer consists of surgery in 10–20% cases when tumor is operable, or chemotherapy with or without radiotherapy in the metastatic setting [1, 4]. Regardless of major efforts, the median survival of patients with metastatic pancreatic cancer remains less than 12 months from diagnosis [1].

Immunotherapy in the form of programmed cell death protein 1 (PD-1) and programmed cell death ligand 1 (PD-L1) inhibitors have revolutionized cancer care in the past decades, in many cases replacing conventional chemotherapy or delivering significant survival improvement when combined with standard therapy [5]. However, pancreatic cancer has remained resistant to PD-1/PD-L1 inhibition, apart from a rare population of pancreatic cancers with high microsatellite instability (MSI-H) representing less than 1% of all patients [6]. Lack of benefit from PD-1/PD-L1 inhibitors in pancreatic cancer is thought to arise from multiple reasons such as lack of immune cell infiltration, their dysfunction, low mutational burden, immunosuppressive tumor microenvironment (TME), and dense stroma limiting drug perfusion [7, 8].

A possible solution to enabling PD-1/PD-L1 inhibitors and re-establishment of immune balance in pancreatic cancer is oncolytic viruses. Oncolytic viruses preferentially replicate in cancer cells, either due to natural selectivity or by genetic modifications, leading to cancer cell death, inflammation and epitope spread [9]. Additionally, oncolytic viruses can be engineered to encode transgenes, leading to high concentrations of cytokines or other cargo at the tumor site, thus functioning as targeted cancer gene therapy and circumventing systemic toxicity [9]. Oncolytic adenoviruses are double stranded DNA viruses that are easy to genetically engineer, exhibit good stability in storage and possess a long history as gene therapy vectors, making them promising vectors for oncolytic virotherapy [10].

Our previous preclinical work demonstrated that combining oncolytic adenovirus encoding tumor necrosis factor alpha (TNF) and interleukin-2 (IL-2) with ICI could significantly improve tumor growth control and survival, even in ICI-resistant and refractory settings [11–13]. In order to avoid expansion of T regulatory cells, we have constructed an oncolytic adenovirus encoding a modified interleukin-2 cytokine with higher preferential binding to interleukin-2 receptor subunit beta, and thus targeting the transgene to resting NK cells and naïve T cells [14]. We hypothesized that using Ad5/3-E2F-d24-vIL2 (“vIL2-virus,” also known as TILT-452), we can direct the early immune response away from an immunosuppressive microenvironment present in many tumors such as PDAC. Previous studies with this virus construct have shown synergism with chemotherapy, NK cell therapy and TIL therapy, supporting the immunostimulatory nature of the virus [15–17].

In the present research, we aimed to study if we could utilize Ad5/3-E2F-d24-vIL2 to enable PD-L1 inhibition in PDAC. In order to maximize the translatability of our research, we studied the combination treatment with standard chemotherapy of PDAC in the form of nanoparticle albumin-bound paclitaxel (nab-paclitaxel) and gemcitabine.

## Materials and methods

### Single cell RNA (scRNA) sequencing analysis

scRNA sequencing analysis of adenovirus serotype 3 primary entry receptors was carried out using a previously published dataset of 25 PDAC fine-needle biopsies and deposited in Gene Expression Omnibus (GSE242230) [18]. scRNA sequencing analysis was conducted with Seurat (version 5.1.0) and RStudio (version 4.3.3) [19]. Cells expressing between 500 and 9000 genes were included in the analysis. Cells with more than 150,000 expressed RNA molecules and more than 25% mitochondrial RNAs of all expressed RNAs were excluded from analysis, along with genes expressed in less than 3 cells. Expression was log-normalized with scale factor of 10,000, principal components (PCs) were produced with 3000 most variable genes, and clustering was done with PC1 to PC40.

### *Cell lines and *in vitro* culture studies*

Human pancreatic cancer cell line Panc-1 (CRL-1469) and human fibroblast cell line MRC-5 (CCL-171) were sourced from ATCC (VA, USA). Human monocyte cell line THP-1 (TIB-1) was a kind gift from Dr. Eija Nissilä and Adj. Prof. Karita Haapasalo. Hamster pancreatic cancer cell line HapT1 was a kind gift from Dr. Ruben Hernandez-Alcoceba. Panc-1 and MRC-5 were grown in DMEM (Biowest, France), THP-1 and HapT1 were grown in RPMI-1640 (Biowest, France). All cell cultures were supplemented with 10% fetal bovine serum (Thermo Fisher Scientific, MA, USA), 1% penicillin–streptomycin (Biowest, France) and 1% L-glutamine (Biowest, France). THP-1 cells were differentiated into M0 macrophages by a protocol described previously using phorbol 12-myristate 13-acetate (Sigma-Aldrich, MO, USA) [20].

For co-culture studies of Panc-1 cells and THP-1 derived macrophages, THP-1 cells were differentiated into M0 macrophages on non-TC treated plates for 24 h, after which media was changed and Panc-1 cells added in 1:1 ratio. For Panc-1 and MRC-5 fibroblast co-cultures, cells were seeded in 1:1 ratio. Co-cultures were treated with viruses at multiplicity of infection (MOI = viral particles per cell = VP/cell) of 100 or interferon gamma (IFNγ, PeproTech, NJ, USA) at concentration of 100 IU/mL 24 h post Panc-1 seeding. Cells for flow cytometry were collected with trypsinization 48 h after infection. For Panc-1 xCELLigence with unmatched PBMCs, 10 000 Panc-1 cell were seeded to xCELLigence plates. After 24 h, unmatched PBMCs were added in an effector to target ratio 10:1, along with treatments (gemcitabine and paclitaxel at 10 nM, virus at 10 MOI and anti-PD-L1 at 20 μg/mL). Cell growth was monitored until 150 h.

### Chemotherapeutics, anti-PD-L1s and viral constructs

For in vitro and ex vivo evaluation, paclitaxel (Sigma-Aldrich, MO, USA) and gemcitabine (Sigma-Aldrich, MO, USA) were used. For ex vivo studies with human PDAC surgical samples, anti-PD-L1 atezolizumab (Roche, Germany) was used. For in vivo experiments, nab-paclitaxel (Celgene, NY, USA), gemcitabine (Accord Healthcare, UK), and a hamster anti-PD-L1 previously developed in our laboratory were used [21].

Viral constructs of Ad5/3-E2F-d24, Ad5/3-E2F-d24-hIL2 and Ad5/3-E2F-d24-vIL2 have been described previously [22]. Transgenes were inserted into the E3 region of the adenovirus genome replacing E3 6.7 K and E3 gp19K using BAC-recombineering [23]. Viruses were produced in A549 cells and purified with cesium chloride centrifugation.

### *Surgical PDAC sample processing and *ex vivo* experiments*

Samples from pancreatic surgery were collected in 10% FBS RPMI, followed with mechanical disruption and incubation in enzyme mix consisting of 5 mg/mL Collagenase XI (Thermo Fisher Scientific, MA, USA) and 10 mg/mL DNAse (Worthington Biochemical, NJ, USA) for 1 h until homogenous suspension formed. Samples were washed twice with 10% FBS RPMI and utilized fresh for downstream assays.

For ex vivo real-time cell analysis experiments, xCELLigence plates (Agilent Technologies, CA, USA) were pre-coated with 10 ug/mL Matrigel (Corning, NY, USA) solution diluted in PBS (Sigma-Aldrich, MO, USA) for 1 h in 4 °C prior to seeding to facilitate tumor cell attachment. After 1 h coating, plates were washed twice with PBS, after which PDAC samples were seeded at 5*10^4^ cells/well. Cell growth was followed until exponential growth started roughly 300 h post-seeding, after which treatments (gemcitabine and paclitaxel at 10 nM, virus at 100 MOI and anti-PD-L1 at 20 μg/mL) were added and cell growth monitored. Cell index was normalized to cell index prior to treatment addition.

For ex vivo cultures, single-cell suspension samples generated as above were seeded into U-bottom 96-well plates at 100,000 cells/well. Cells were treated 24 h post-seeding with Ad5/3-E2F-d24-vIL2 (100 MOI) or anti-PD-L1 (20 μg/mL), or combination, and supernatant and cells were collected for further analysis prior to flow cytometric analysis.

### Proteomic and flow cytometric analysis

Supernatant proteomic analysis was conducted with LEGENDplex Human Essential Immune Response Panel kit (Biolegend, CA, USA) according to manufacturer’s recommendations. Cytokine amounts were normalized to mock treated values. Flow cytometry staining was conducted after blocking either with human Fc-block (BD Biosciences, NJ, USA) or mouse Fc-block (BD Biosciences, NJ, USA). Flow cytometric antibodies used are listed in Supplementary Table 1. Minimum 2*10^4^ events were collected per sample using NovoCyte Quanteon cytometer (Agilent Technologies, CA, USA). Compensation was conducted with UltraComp eBeads (Thermo Fisher Scientific, MA, USA) according to manufacturer’s recommendations. Exported FCS-files were analyzed with FlowJo v10.10.0 (BD Biosciences, NJ, USA).

### qPCR for viral genomes

qPCR was conducted with primers targeting the adenoviral hexon, with normalization to human beta-actin gene amounts. Primer and probe sequences are shown in Supplementary Table 2. Samples were run in duplicate, quantified using a normal curve and an average of two samples was used for downstream analysis.

### Hamster PDAC model and treatments

For in vivo validation, female Golden hamsters (*Mesocricetus auratus*, RjHan:AURA, Janvier Labs, France) aged 6 weeks were used. 10^6^ HAPT1 cells were engrafted subcutaneously to the left flank of the animals, and after mean tumor growth of 100 mm^3^, animals were randomized to treatment groups (N = 8 per group) of mock treatment, chemotherapy, chemotherapy + anti-PD-L1, or chemotherapy + anti-PD-L1 + virotherapy. Animals received nab-paclitaxel intraperitoneally at a dose of 30 mg/kg once weekly, gemcitabine intraperitoneally at a dose of 60 mg/kg once weekly, anti-PD-L1 intraperitoneally at a dose of 300 ug once weekly, and Ad5/3-E2F-d24-vIL2 at a dose of 10^9^ viral particles (VPs) intratumorally twice weekly. All animals not receiving virus received intratumoral PBS treatment as control. Treatment was given for 4 rounds of chemotherapy and 8 rounds of virotherapy, after which no further treatment was administrated. Animals were monitored for adverse effects through visual observation and weight tracking, and they were euthanized if signs of unacceptable toxicity were detected, defined as loss of bodyweight more than 20% or visual suffering exhibited by the animal. Tumor growth was measured with digital calipers three times a week. Animals were euthanized when longest tumor diameter (length) reached 22 mm. Tumor volume was calculated as (width^2^ × length)/2 and normalized to volume at the start of the treatment. A non-terminal tumor biopsy was collected on day 9 following protocol published previously [24].

For re-challenge in vivo experiment, animals cured by the treatment were engrafted with original HAPT1 cell line and non-encountered leiomyosarcoma cell line DDT1-MF2. A control group of six-week-old naïve animals was used. 2 million cells of each cell line were engrafted to the upper flank, and HAPT1 was engrafted contralaterally to the original tumor location. Animal received no additional treatment, and tumor growth was measured every two days. All animals were euthanized on day 12 when tumor diameter reached 22 mm in the first animal. Spleens were collected and mechanically disrupted by passing through 70 um filter, followed with ACK (Thermo Fisher Scientific, MA, USA) lysis and flow cytometric analysis.

### Statistical analysis

For grouped analyses, samples were compared with unpaired Welch *t*-test. Four parameter logistic (4PL) sigmoidal curve was used for gemcitabine and paclitaxel dose–response to viral replication. For tumor growth comparison, a mixed-effects model was used. For survival analysis, logrank test was used. A *p*-value less than 0.05 was considered statistically significant.

### Ethical permission

Ethical permission for clinical PDAC sample collection and analysis was approved by the Helsinki University Hospital Ethical Board (HUS/2761/2021). All patients gave consent for sample collection and analysis. Animal experimentation was reviewed and approved by the Regional State Administrative Agency of Southern Finland (ESAVI/26562/2022).

## Results

### Oncolytic adenovirus and chemotherapy upregulate PD-L1 on cancer cells and gemcitabine negatively impacts viral replication

**Fig. 1:**
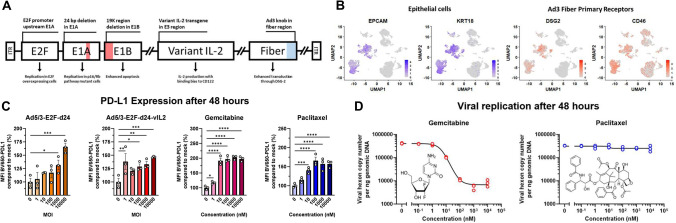
**A** Genomic structure of Ad5/3-E2F-d24-vIL2. **B** scRNAseq analysis of Ad3 primary receptor localization to epithelial (cancer) cells in previously published dataset of 25 PDAC biopsies. **C** PD-L1 expression on Panc-1 after 48 h of infection with different MOIs of unarmed or variant IL-2 virus, or treatment with gemcitabine or paclitaxel, groups compared with two-sided unpaired t test. **D** Effects of different concentrations of gemcitabine and paclitaxel on viral replication by qPCR. Curves fitted with 4PL sigmoidal function. ITR = inverted terminal repeat, KRT18 = keratin 18, DSG2 = desmoglein-2, MFI = median fluorescence intensity, MOI = multiplicity of infection in VP/cell, GEM = gemcitabine, PTX = paclitaxel, * = *p* < 0.05, ** = *p* < 0.01, *** = *p* < 0.001, **** = *p* < 0.0001. Number of biological replicates = 3 for C-D, number of technical replicates = 1 for C, 2 for D

An oncolytic adenovirus encoding a modified IL-2 molecule with preferential binding to IL-2RB (CD122) was constructed by adding the transgene into the adenoviral E3 region (Fig. 1A). Cancer cell specificity was achieved with E2F promoter upstream of 24 bp deleted E1A region, making the virus double-specific for mutated p16/Rb/E2F pathway, widely present in cancer [25]. Additionally, the virus harbors a deletion in the adenoviral E1B region encoding the E1B-19K protein, enhancing cancer cell apoptosis, and a change in the adenovirus serotype 5 knob to adenovirus serotype 3 knob, facilitating transduction of most solid cancers through desmoglein-2 (DSG2) [26, 27].

To assess if our viral construct would be a suitable vector against PDAC, we re-analyzed a previously published dataset of scRNA sequencing from 25 PDAC fine-needle biopsies [18]. In analyzed samples, primary Ad3 fiber receptors DSG2 and CD46 localized to epithelial cells defined as epithelial cell adhesion molecule (EPCAM) or keratin 18 (KRT18) positive cells (Fig. 1B), supporting the feasibility of the vector.

As this study focused on enabling PD-1/PD-L1 inhibition in PDAC, we next assessed the effects of virotherapy and chemotherapy on PD-L1 expression on pancreatic cancer cells, Panc-1 cells were infected with unarmed virus, virus armed with normal human IL-2 (Ad5/3-E2F-d24-hIL2) and Ad5/3-E2F-d24-vIL2. All viruses significantly increased PD-L1 expression on cancer cells 48 h post infection in a dose dependent manner (Fig. 1C, Supplementary Fig. 1A). Similarly, both gemcitabine and paclitaxel increased PD-L1 expression on cancer cells (Fig. 1C. No increase in secreted PD-L1 could be seen in the supernatant following treatment with virotherapy, chemotherapy or in combination (Supplementary Fig. 1B). No clear dose dependency could be observed for PD-L2 (Supplementary Fig. 1C). No clear change in cell morphology or DAPI staining could be seen with any viral dose (Supplementary Fig. 1D).

To study if gemcitabine or paclitaxel inhibited viral replication, we co-treated Panc-1 cells with increasing doses of chemotherapy and a constant dose of Ad5/3-E2F-d24-vIL2. Increasing concentrations of gemcitabine inhibited viral replication roughly 50-fold (Fig. 1D) but increasing doses of paclitaxel did not have measurable effect on viral replication (Fig. 1D).

### Cancer cell, fibroblast and macrophage co-culture infection leads to modulation of PD-L1 expression and other B7 family protein expression

**Fig. 2:**
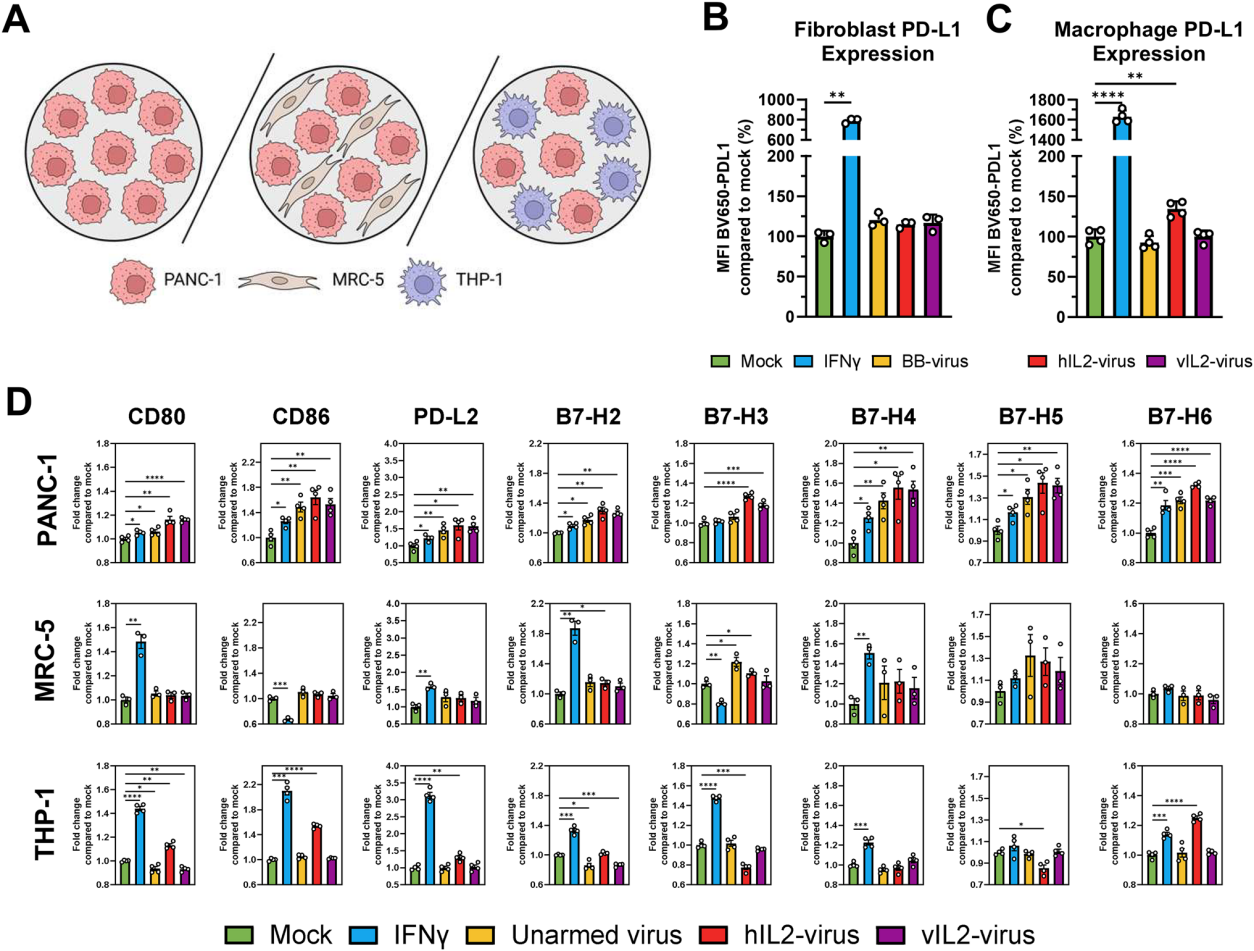
**A** Schematic for co-culture experiments. **B** PD-L1 expression on fibroblasts (MRC-5) after 48 h of infection in co-culture. **C** PD-L1 expression on macrophages (THP-1) after 48 h of infection in co-culture. **D** Expression fold changes of other B7 family proteins in cancer cells, fibroblasts or macrophages. Fold change calculated from average of mock. MOI = VP/cell = 100. For B-D, groups compared with two-sided unpaired *t*-test, MFI = median fluorescence intensity, * = *p* < 0.05, ** = *p* < 0.01, *** = *p* < 0.001, **** = *p* < 0.0001. Number of biological replicates = 3 for B-C, 3–4 for D

Pancreatic cancer is known to have high stromal content, with high abundance of immunosuppressive cell types. To gain a more in-depth knowledge of the effects of virotherapy on PD-L1 and other B7 family proteins, we infected monocultures of Panc-1 and co-cultures of Panc-1 with fibroblasts (MRC-5) or macrophages (THP-1 derived macrophages) with unarmed virus, virus producing normal human IL-2 and Ad5/3-E2F-d24-vIL2 (Fig. 2A). IFNγ was used as a positive control due the known increasing effect on PD-L1. When assessing PD-L1 expression on fibroblasts, no significant increase could be seen after viral treatment, possibly suggesting that cancer cell PD-L1 expression is tied to complete viral replication (Fig. 2B). However, virus producing normal human IL-2 did induce PD-L1 expression on macrophages (Fig. 2C), possibly due to normal IL-2 being able to bind IL-2 receptors on macrophages.

Regarding other B7-family proteins, increased expression of most other B7 family proteins was seen in Panc-1 cells, supporting that viral replication leads to increased expression of these proteins (Fig. 2D, first row). However, when assessing B7 family proteins on fibroblasts, significant increases could only be seen for B7-H2 and B7-H3 after viral infection (Fig. 2D, second row). Regarding B7 family expression on macrophages, normal human IL-2 encoding virus led to increased expression of CD80, CD86, PD-L2 and B7-H6 and downregulation of B7-H3 and B7-H5 (Fig. 2D, bottom row). Unarmed virus and variant IL-2 virus produced modest effects, with downregulation of B7-H2 being observed, suggesting that binding of the normal human IL-2 protein likely was driving the effects seen in macrophages.

### Combining Ad5/3-E2F-d24-vIL2 with anti-PD-L1 and chemotherapy leads to synergistic killing of clinical PDAC ex vivo samples

**Fig. 3:**
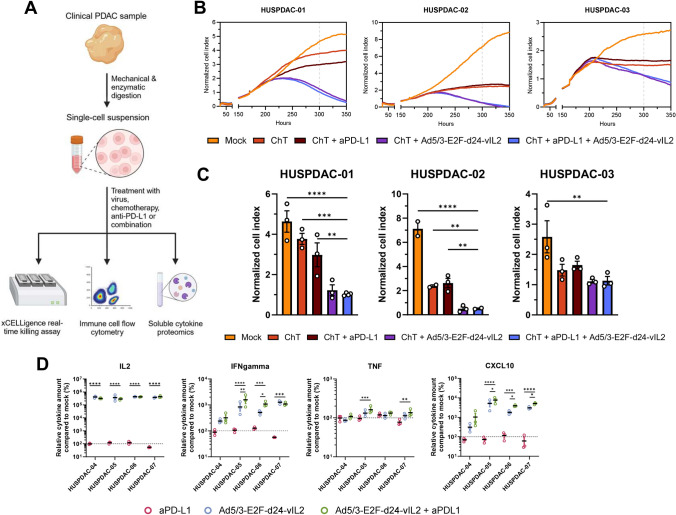
**A** Schematic for sample processing. **B** xCELLigence real-time cell killing assay of HUSPDAC-01, HUSPDAC-02 and HUSPDAC-03. **C** Cell viability at 300 h comparing in all groups. **D** Expression of IL-2, IFNγ, TNF-alpha and CXCL10 in supernatant of histocultures of HUSPDAC-04, HUSPDAC-05, HUSPDAC-06 and HUSPDAC-07. For C-D, groups compared with unpaired *t*-test, ChT = chemotherapy (paclitaxel + gemcitabine), * = *p* < 0.05, ** = *p* < 0.01, *** = *p* < 0.001, **** = *p* < 0.0001. Number of biological replicates = 3 for B-D

To study the combination of virotherapy with anti-PD-L1 therapy and chemotherapy in a clinically relevant setting, we collected seven pancreatic ductal adenocarcinoma samples fresh from surgery. Samples collected and patient characteristics are shown in Table 1. Following sample collection, samples were dissociated mechanically and enzymatically, and used fresh for downstream assays (Fig. 3A). Utilizing xCELLigence real-time killing assay, we evaluated samples HUSPDAC-01 to HUSPDAC-03 treated with mock, chemotherapy, chemotherapy + anti-PD-L1, chemotherapy + Ad5/3-E2F-d24-vIL2 or chemotherapy + anti-PD-L1 + Ad5/3-E2F-d24-vIL2. All three samples showed significantly enhanced killing in groups where Ad5/3-E2F-d24-vIL2 was present (Fig. 3B, C). Depending on the sample, anti-PD-L1 alone had minimal effect on cell killing, mimicking the effects seen in the clinic.

**Table 1:** Patient demographics of collected clinical samples

Sample	Sex	Age (years)	Histology	TNM	Molecular status	Neo-adjuvant treatment	Location of tumor in pancreas
HUSPDAC-01	F	70	PDAC	pT2N0	MSS	–	Body
HUSPDAC-02	F	79	PDAC	pT2N2	–	–	Head
HUSPDAC-03	M	65	PDAC	pT2N2	–	–	Head
HUSPDAC-04	F	60	PDAC	pT4N2	–	NPTX + GEM	Head
HUSPDAC-05	F	70	PDAC	pT2Nl	–	–	Head
HUSPDAC-06	F	78	PDAC	pT3Nl	–	–	Head
HUSPDAC-07	F	71	PDAC	pTlcNl	–	–	Head

*F* female, *M *male, *PDAC *pancreatic ductal adenocarcinoma, *MSS* microsatellite stable, *NPTX + GEM* nab-paclitaxel + gemcitabine

Concomitantly, we utilized samples HUSPDAC-04, HUSPDAC-05, HUSPDAC-06 and HUSPDAC-07 for ex vivo cultures. Proteomic profiling of the supernatants showed active IL-2 production in the samples, supporting active replication of the vector (Fig. 3D). Furthermore, proteomic analysis highlighted possible synergism behind combining PD-L1 inhibition to virotherapy, since groups treated with the combination of anti-PD-L1 and Ad5/3-E2F-d24-vIL2 showed significantly enhanced secretions of IFNγ, TNF and CXCL10 (Fig. 3D). Interestingly, sample HUSPDAC-04 from a patient that had received neo-adjuvant chemotherapy prior to surgery showed markedly less pronounced secretion of IFNγ and CXCL10, indicating that the neo-adjuvant chemotherapy had affected the immune cells in the sample (Fig. 3D). However, no difference in IL-2 was seen, suggesting that neo-adjuvant chemotherapy had little effect on viral replication (Fig. 3D). Other cytokines assayed from the samples showed only marginal changes and are shown in Supplementary Fig. 3.

We further validated the findings with an additional experimental group utilizing Panc-1 cell and unmatched PBMCs in an effector to target ratio of 10:1. The assay results confirmed superior killing with groups receiving chemotherapy combined to checkpoint inhibition and virotherapy. Interestingly, vIL2-virus group not receiving chemotherapy showed markedly poor cancer cell killing, highlighting the importance of all three components in the combination treatment (Supplementary Fig. 2). Additionally, the unarmed virus exhibited potent cell killing even without chemotherapy. We have also previously noted and published regarding the ability of the unarmed virus to more efficiently kill Panc-1 cells [15].

### Ad5/3-E2F-d24-vIL2 treatment leads to expansion of cytotoxic T cells in PDAC histocultures with increased PD-1 expression

**Fig. 4:**
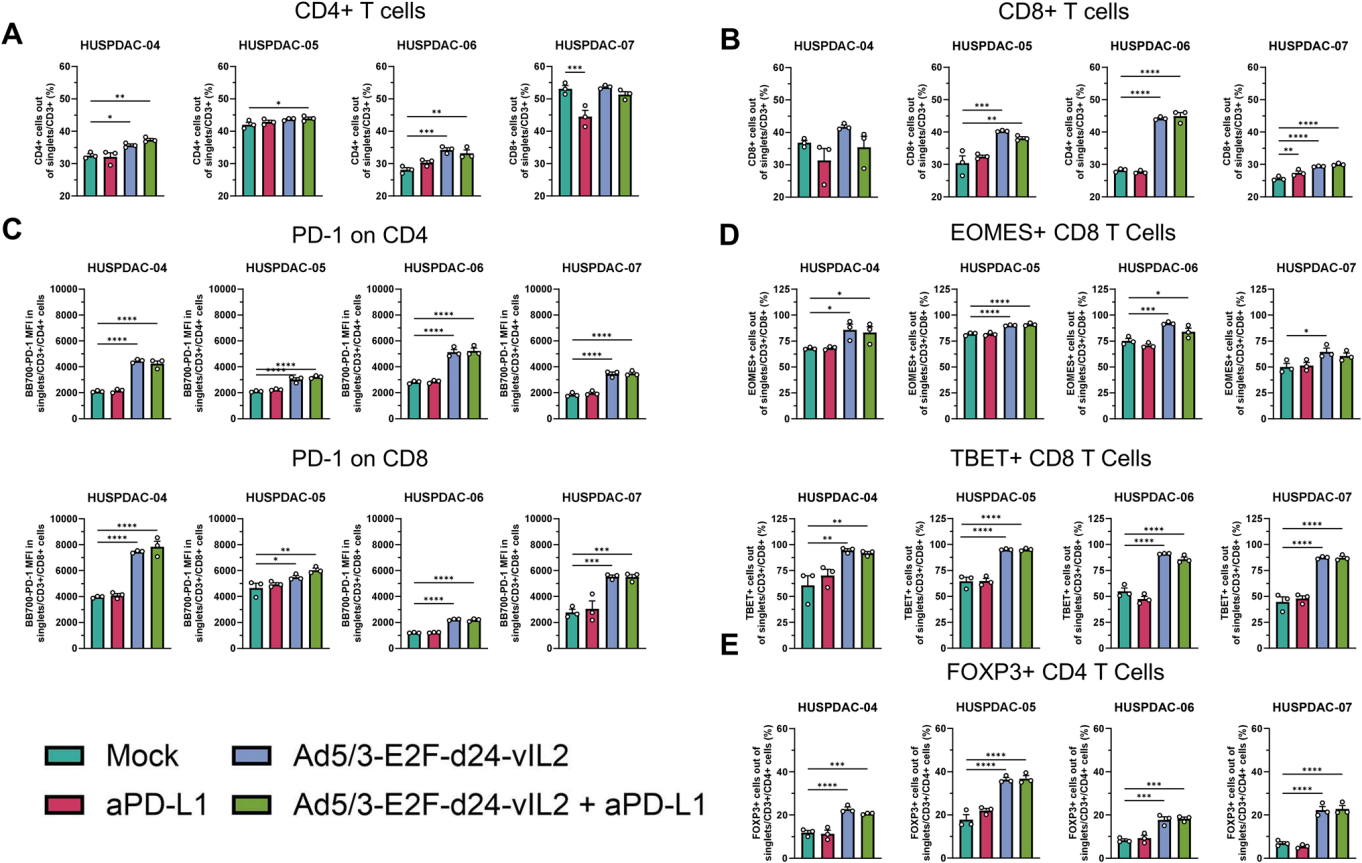
**A** Changes in CD4 + cell amount in histocultures of HUSPDAC-04, HUSPDAC-05, HUSPDAC-06 and HUSPDAC-07. **B** Changes in CD8 + cell amount in histocultures of HUSPDAC-04, HUSPDAC-05, HUSPDAC-06 and HUSPDAC-07. **C** Changes in PD-1 expression on CD4 + and CD8 + cells in histocultures of HUSPDAC-04, HUSPDAC-05, HUSPDAC-06 and HUSPDAC-07. **D** Changes in EOMES and T-BET expression in CD8 + cells in histocultures of HUSPDAC-04, HUSPDAC-05, HUSPDAC-06 and HUSPDAC-07. **E** Changes in CD4 + FOXP3 + T cells in histocultures of HUSPDAC-04, HUSPDAC-05, HUSPDAC-06 and HUSPDAC-07. Groups compared with two-sided unpaired *t*-test, * = *p* < 0.05, ** = *p* < 0.01, *** = *p* < 0.001, **** = *p* < 0.0001. Number of biological replicates = 3

In addition to proteomic profiling, we studied immune cells present in the histocultures. Flow cytometric profiling of patients HUSPDAC-05 to HUSPDAC-07 showed increased amounts of both CD4 + and CD8 + T cells in the treated samples (Fig. 4A, B). Additionally, increase in PD-1 expression, the receptor for PD-L1, was seen on both CD4 + and CD8 + T cells (Fig. 4C), providing rationale to circumvent possible inhibitory effects by blocking PD-L1 binding. To assess the activation status of CD8 + cells, we assessed the effector transcription factors eomesodermin (EOMES) and T-bet, which are crucial for anti-tumor T cell responses [28]. In samples treated with Ad5/3-E2F-d24-vIL2, significant upregulation of both EOMES and T-bet could be seen, supporting that the cells were activated. Due to the design of the variant IL-2 molecule with the aim of not expanding T regulatory cells, we also studied these cells in the ex vivo cultures. Interestingly, all assayed samples showed significant increases in CD4 + FOXP3 + cells, possibly arising from normal IL-2 secretion from CD4 + T cells present in the samples (Fig. 4E). To confirm this, we performed an ex vivo PBMC expansion experiment, utilizing healthy donor PBMCs stimulated with anti-CD3 antibody, and either recombinant human IL-2, viral supernatant from Panc-1 infected with Ad5/3-E2F-d24-hIL2 or Ad5/3-E2F-d24-vIL2. Results from this experiment showed expansion of FOXP3 + CD4 cells in all scenarios, although the supernatant from Ad5/3-E2F-d24-vIL2 group showed the highest CD8 to FOXP3 ratio, and highest CD8 to CD4 ratio (Supplementary Fig. 4A-B).

### In vivo validation shows enhanced tumor growth control and survival of triple combination therapy mediated by increased immune cell infiltration

**Fig. 5:**
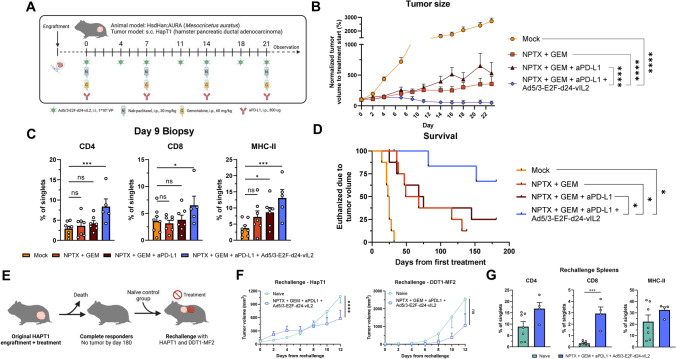
**A** Schematic of animal experiment. **B** Tumor size in animals normalized to baseline. **C** Changes in intratumoral CD4 +, CD8 + and MHC-II cells in day 9 tumor biopsies. **D** Survival analysis of animals. **E** Re-challenge experiment schematic. **F** Tumor growth of original HapT1 cell line and new DDT1-MF2 cell line. **G** Flow cytometry of CD4 +, CD8 + and MHC-II + cells in re-challenge spleens. For B and F, groups compared with mixed-effects analysis. For C and G, groups compared with two-sided unpaired *t*-test. For D, groups compared with logrank test. * = *p* < 0.05, *** = *p* < 0.001, **** = *p* < 0.0001. NPTX = nab-paclitaxel, GEM = gemcitabine

Next, we aimed to assess the effects of our treatment in a proof-of-concept in vivo experiment. Due to inability of human adenoviruses to replicate in mouse tumors, we utilized Golden hamsters as an in vivo model, since human adenoviruses replicate semipermissively in these animals [29]. The experimental scheme is shown in Fig. 5A.

Triple combination treatment showed significantly improved tumor growth control compared to all other treatment groups (Fig. 5B). We performed a non-terminal tumor biopsy on day 9 to study immune changes in tumors with flow cytometry. Samples showed increased amounts of CD4 + , CD8 + and MHC-II + cells, indicating that virotherapy is able to recruit immune cells into tumors and/or support their proliferation (Fig. 5C). Follow-up of animals showed that triple therapy also translated to significant survival benefit, whereas PD-L1 therapy alone had minimal survival benefit, again mimicking the situation seen in the clinic (Fig. 5D).

### Animals cured by the triple combination therapy exhibit enhanced protection from re-challenge

After follow-up for 180 days, animals without tumors were utilized for re-challenge experiment, with a control group of naïve animals without prior experience to tumors. Animals were challenged with the original cell line HapT1 and unencountered leiomyosarcoma cell line DDT1-MF2 (Fig. 5E). Animals cured by the triple therapy showed significantly improved tumor control of the original HapT1 cell line, however no significant protection advantage for DDT1-MF2 could be seen (Fig. 5F).

In order to study systemic immune changes, spleens of re-challenged animal were collected and analyzed. Spleens of triple therapy animals showed significantly more CD8 + cells, with non-significant increases in CD4 + and MHC-II + also present, supportive of generation of systemic anti-cancer immunity following chemotherapy, ICI and virotherapy (Fig. 5G).

## Discussion

The resistance of PDAC to immunotherapy remains a significant challenge. While PD-1 and PD-L1 inhibitors are standard treatments for many solid tumors, PDAC has shown resistance to these therapies. In this study, we showed that anti-PD-L1 therapy can be enabled in different PDAC models by utilizing an oncolytic adenovirus encoding an immunostimulatory transgene. Ad5/3-E2F-d24-vIL2 in combination with anti-PD-L1 showed synergistic activation of T cells in clinical PDAC samples, and an in vivo animal experiment showed that treatment with Ad5/3-E2F-d24-vIL2 leads to increased amounts of CD8 + and CD4 + T cells infiltrating the tumors, thus correcting known causes of poor response of PDAC to immunotherapy. Indeed, it was reported already 20 years ago that immune infiltration influences survival of pancreatic cancer patients, with more recent reports utilizing more sophisticated research tools linking long survival to T cell polyclonality and higher neoantigen presence [30–32]. Importantly, both T cell infiltration and neoantigen presence have been identified as key for patient survival, and neither attribute alone is enough to drive a survival benefit [30]. Recent reports of successes in mRNA vaccine therapy have highlighted the possibility of utilizing immunotherapy for treating PDAC and achieving durable cures in the neo-adjuvant setting [33]. Oncolytic virotherapy is a compelling solution to facilitate long survival in PDAC, due to its ability to induce both T cell infiltration and antigen spreading in tumors [9].

We profiled the effects of Ad5/3-E2F-d24-vIL2 in different PDAC models. Through scRNA of previously published biopsy data, we showed that PDAC exhibits receptors for vector entry [18]. Additionally, we showed that viral replication in cancer cell line Panc-1 leads to increased PD-L1 expression on the cell surface, a finding consistent with previous reports and supporting the rationale of the combination therapy [34, 35]. Previous research has identified PD-L1 expression increase following infection with other types of oncolytic viruses [35]. Other research has identified IFN-alpha and IFN-beta as possible mediators of PD-L1 expression, and this is possibly applies also for adenoviruses since we saw PD-L1 upregulation in monoculture studies [36]. Additionally, we showed that treatment with paclitaxel or gemcitabine led to increased PD-L1 expression on cancer cells, highlighting the importance of the pathway. We also showed that PD-L1 expression seems to be linked to viral replication, since neither fibroblasts or macrophages showed upregulated PD-L1 in co-culture settings with Ad5/3-E2F-d24-vIL2 treatment. However, macrophages did upregulate PD-L1 when co-cultures were treated with an oncolytic adenovirus producing an unmodified version of interleukin-2, suggesting that choice of cytokine can be important when designing oncolytic adenoviruses. Macrophages are known to express IL-2 receptors, and interleukin-2 has been known to activate macrophages, especially when interferon gamma is present [37, 38]. Our previous studies comparing normal IL-2 producing virus to variant IL-2 producing virus showed that in vivo, markers of suppressive myeloid cells are not expanded with variant IL-2 virus, in comparison with normal IL-2 producing virus which increased both antigen presenting and suppressive myeloid cells [39].

Furthermore, we characterized effects of different virus constructs on other B7 family proteins, highlighting the dynamic changes seen in this important group of proteins, providing valuable information for future combinatory immunotherapeutic studies. We identified that viral effects on B7-family proteins are mostly confined to the infected cancer cells in the co-culture setting, most likely due to the effects being tied to viral replication. Interestingly, we did note that B7-H3 expression was upregulated in cancer cells only after infection with armed adenoviruses, but not with the unarmed virus. Although further research is needed, this effect could be linked to the E3 region being deleted for genes encoding E3 6.7K and E3 gp19K, which have known immunomodulatory effects relating to apoptosis and MHC-I presentation [40–42]. B7-H3 is a known effector of immune escape and helps cancers cells evade killing by T cells and NK cells, and is a marker of poor prognosis in many cancers, similarly to PD-L1 [43–47]. Active studies are ongoing to develop monoclonal antibodies, chimeric antigen receptor T cells and antibody–drug conjugates against B7-H3, with multiple trials in phase I and II and results awaited [48]. Importantly, virus treatment upregulated also other members of the B7 family, many of which are also under therapeutic development and thus possible synergistic future combination therapeutics [49].

Our ex vivo experiments utilizing fresh samples from PDAC surgery highlighted the synergy behind combination of virotherapy with PD-L1 inhibition, resulting in efficient cell killing and cytokine expression. Similarly, Ad5/3-E2F-d24-vIL2 therapy was able to expand T lymphocyte populations in the samples, and interestingly an increase in T regulatory cells was also seen, most likely due to regular IL-2 produced by activated T lymphocytes in the samples*.* We have previously observed this same phenomenon when studying Ad5/3-E2F-d24-vIL2, and in the original report of the vector we showed that roughly 50% of IL-2 or vIL2 in tumors in virus derived, whereas the other 50% is endogenous IL-2 likely produced by CD4 T cells [39]. Interestingly, even though we showed that gemcitabine chemotherapy decreased viral replication by roughly 1.5 logs, this did not seem to inhibit cell killing in the ex vivo experiments utilizing clinical pancreatic surgery samples. This could be due to previously reported synergism of gemcitabine and oncolytic adenoviruses, where gemcitabine does slow replication of the virus, but leads to enhanced toxicity to cancer cells due to combined cytotoxic effects of gemcitabine and the virus [50, 51]. Additionally, other independent research has reported that adenoviral E1A protein can sensitize hepatocellular carcinoma cells to gemcitabine [52]. An in vivo experiment testing triple therapy in Golden hamster confirmed the proposed beneficial effects of the therapy: animals treated with the triple therapy showed increased intratumoral lymphocytes, best tumor control and longest survival. Additionally, animals cured with the therapy showed resistance to tumor re-engraftment, supporting that treatment was able to generate long term anti-tumor memory in the cured animals.

Our research has multiple limitations. Due to limited tissue sample size, we had to omit multiple experimental groups, such as ones containing unarmed virus or normal human IL-2 encoding virus in settings where clinical pancreatic cancer samples were used. Additionally, in the in vivo experiment we focused the experimentation to assess if virotherapy would be able to enable checkpoint inhibition in the chemotherapy setting, as most metastatic pancreatic cancer patients receive chemotherapy as first-line treatment. We have published previous research showing superiority of Ad5/3-E2F-d24-vIL2 to unarmed and normal human IL-2 encoding virus, but the generalizability of the monotherapy results to settings where combination therapies are used are to be determined [39]. Additionally, although utilization of fresh pancreatic cancer samples increase translatability of findings to humans, the pancreatic samples did go through extensive processing in the laboratory and were cultured in nutrient rich mediums such as DMEM. This is in comparison to thick stroma present in pancreatic tumors, in addition to the nutrient poor and hypoxic TME [8, 53]. Furthermore, as the in vivo experiment aimed to only assess the ability of Ad5/3-E2F-d24-vIL2 to potential anti-PD-L1 treatment in clinically relevant treatment schemes which contain chemotherapy as a backdrop, no groups receiving only Ad5/3-E2F-d24-vIL2 or Ad5/3-E2F-d24-vIL2 combined with chemotherapy were included. However, we have previously reported superiority of Ad5/3-E2F-d24-vIL2 combined to chemotherapy over solely chemotherapy [15]. Finally, due to limited clinical sample sizes discussed above, in addition to limitations in the sample collection in the in vivo experiment, no assessment for PD-L1 expression was done in the ex vivo or in vivo settings. We have previously shown that oncolytic adenovirus constructs with similar backbone induce PD-L1 expression in vivo, but we did not assess the situation in the current research setting [54, 55].

Findings in this manuscript demonstrate a promising therapeutic modality for the treatment of metastatic PDAC. Our triple combination therapy represents a clinically testable enhancement of routine nab-paclitaxel and gemcitabine, for improving treatment results in metastatic PDAC.

## Supplementary Information

Below is the link to the electronic supplementary material.

Supplementary file1 (PDF 1062 kb)

Supplementary file2 (PDF 114 kb)

Supplementary file3 (PDF 71 kb)

Supplementary file4 (XLSX 12 kb)

## Data Availability

Data are provided within the manuscript or supplementary information files.
