# Supplementary material for: Oncolytic adenovirus encoding variant interleukin-2 combined with chemotherapy enables PD-L1 inhibition in pancreatic cancer models
Source: Cancer Immunol Immunother. 2025 Jun 4;74(7):234. doi: 10.1007/s00262-025-04072-6 (PMC12137825; doi:10.1007/s00262-025-04072-6)
Supplement: Supplementary file 1 — Supplementary file1 (PDF 1062 kb) [file 262_2025_4072_MOESM1_ESM.pdf]

Oncolytic adenovirus encoding variant interleukin 2 combined with chemotherapy enables PD-L1 inhibition in pancreatic cancer models

Supplementary figures

Supplementary Figure 1

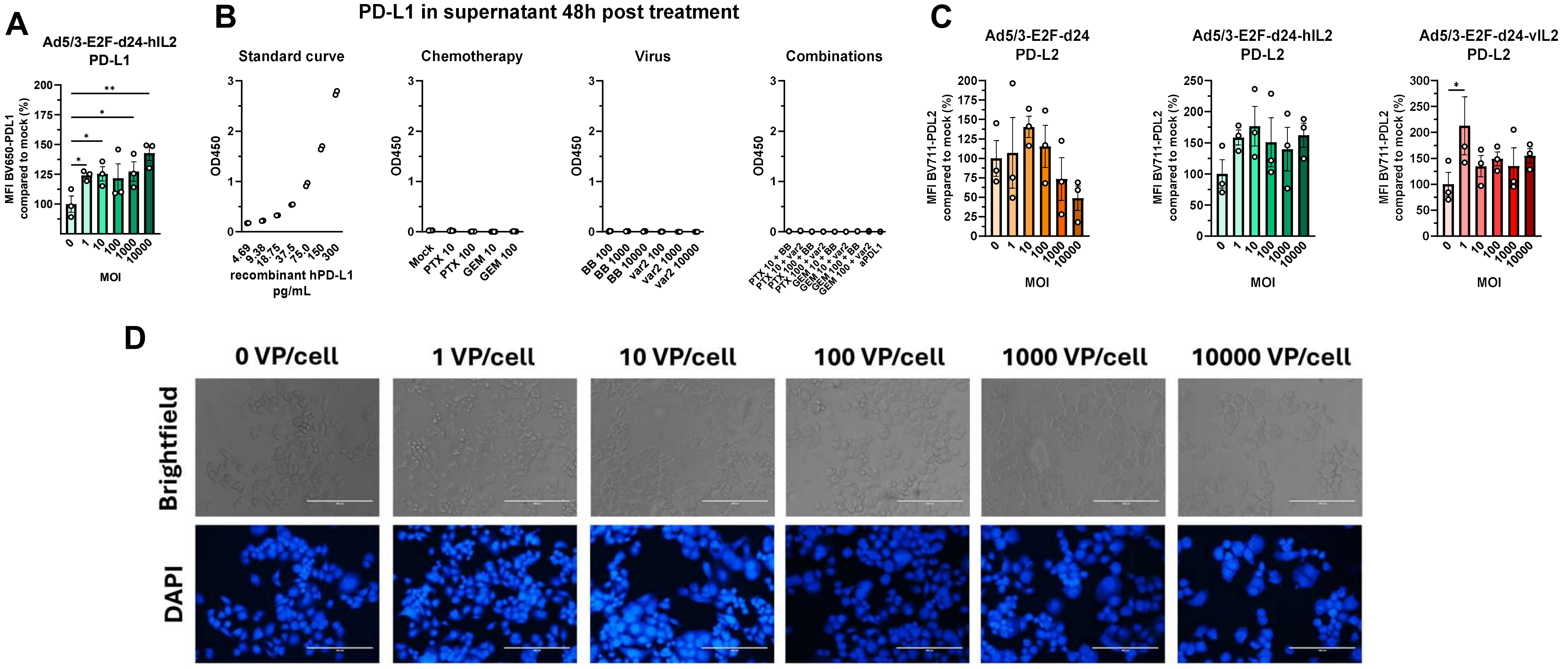

Supplementary Figure 1. A. PD-L1 expression at 48 hours on Panc-1 cell after Ad5/3-E2F-d24-hIL2 infection. **B.** PD-L1 detection in supernatant of Panc-1 after infection with backbone virus, vIL2-virus or treatment with chemotherapy or chemotherapy and virotherapy combination. **C.** PD-L2 expression at 48 hours on Panc-1 cell after Ad5/3-E2F-d24, Ad5/3-E2F-d24-hIL2 and Ad5/3-E2F-d24-vIL2 infection. **D.** Panc-1 cells infected for 48 hours with Ad5/3-E2F-d24-vIL2, fixed with 4% PFA, permeabilized with 100% ice-cold methanol and stained with DAPI. Groups compared with two-sided unpaired t-test, * = p < 0.05, ** = p < 0.01.

Supplementary Figure 2

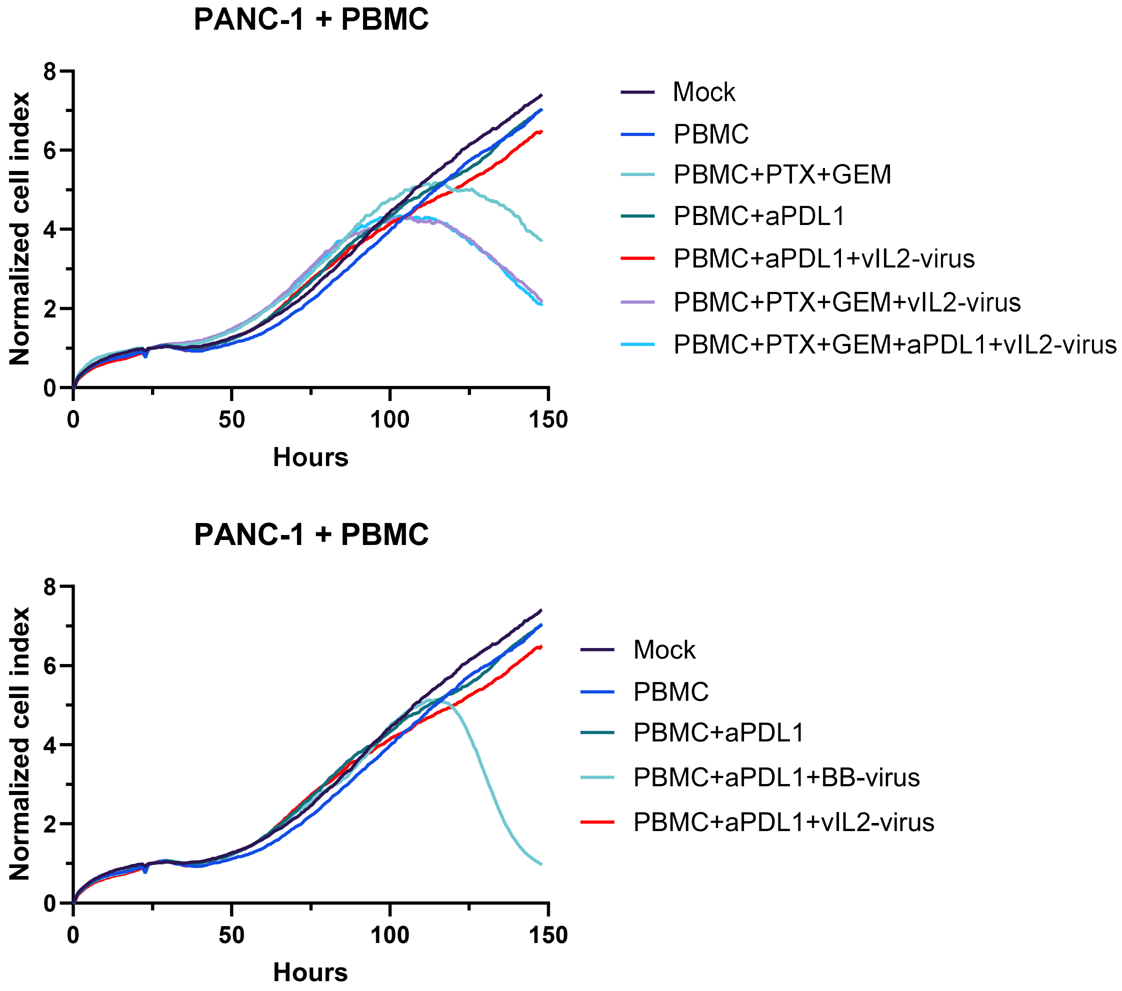

Supplementary Figure 2. PANC-1 co-culture with unmatched PBMCs at effector to target ratio of 1:10 and different treatment groups. N=2 biological replicates, mean shown.

Supplementary Figure 3

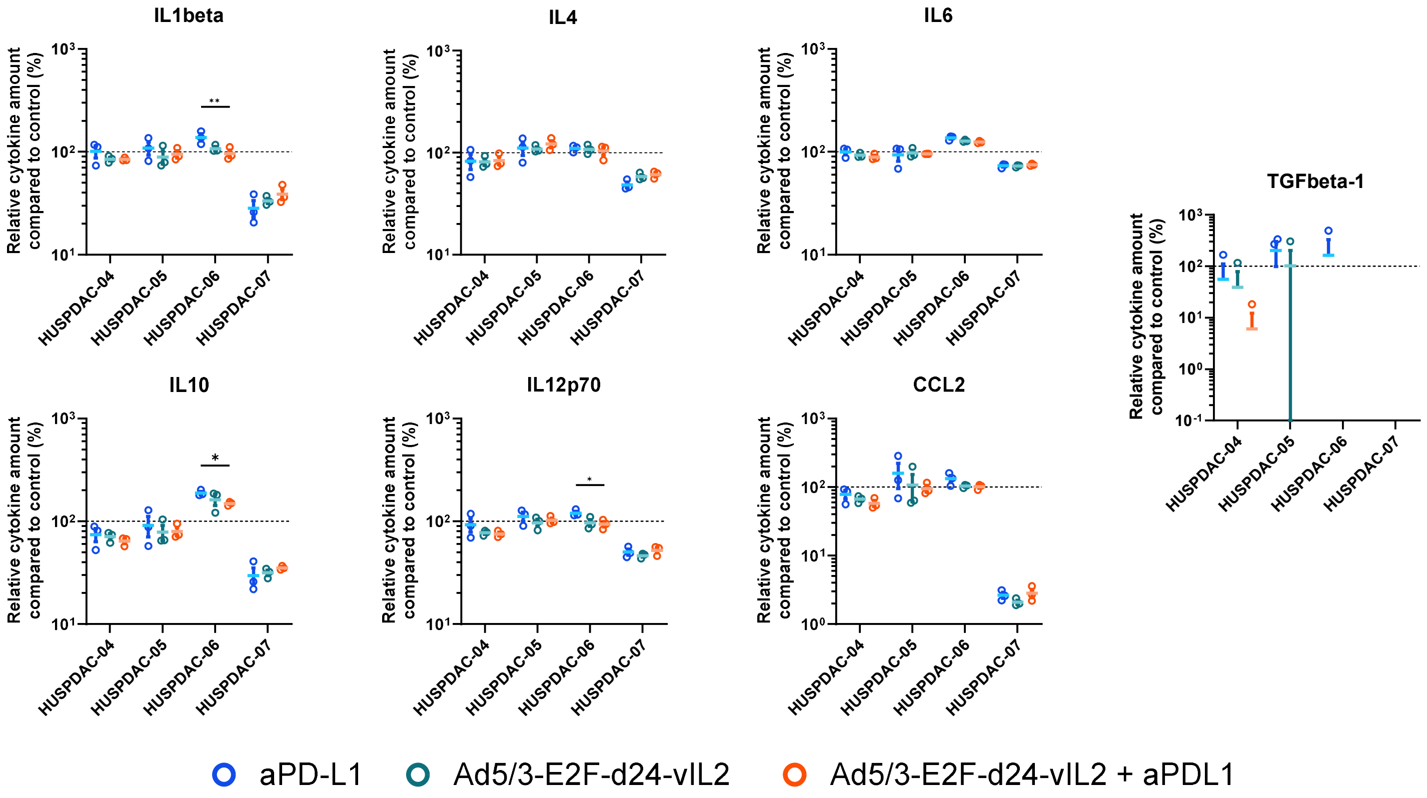

Supplementary Figure 2. Cytokine measurement in histocultures of HUSPDAC-04, HUSPDAC-05, HUSPDAC-06, and HUSPDAC-07. Groups compared with two-sided unpaired t-test, * = p < 0.05, ** = p < 0.01.

Supplementary Figure 4

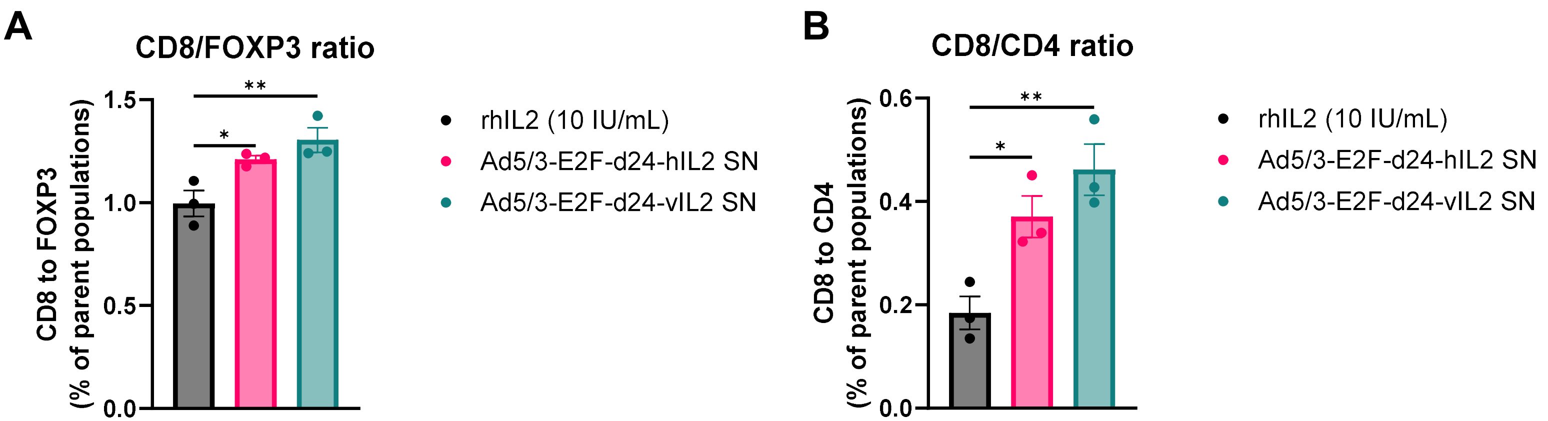

Supplementary Figure 4. Healthy donor PBMCs stimulated with anti-CD3 and supplemented with either recombinant human IL-2, supernatant from Ad5/3-E2F-d24-hIL2 or Ad5/3-E2F-d24-vIL2 for 96 hours. **A.** CD8 to FOXP3 ratio following expansion. **B.** CD8 to CD4 ratio following expansion. Groups compared with two-sided unpaired t-test, * = $p < 0.05$, ** = $p < 0.01$.

Supplementary Figure 5

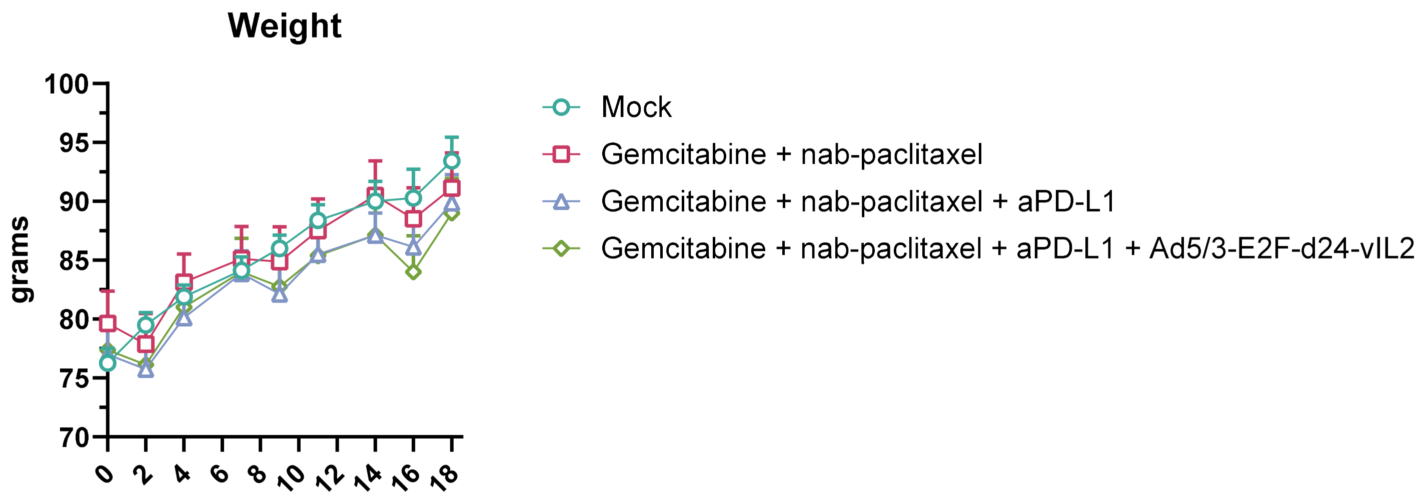

Supplementary Figure 5. Weight change in in vivo experiment.
