# Supplementary material for: Oncolytic adenovirus encoding variant interleukin-2 combined with chemotherapy enables PD-L1 inhibition in pancreatic cancer models
Source: Cancer Immunol Immunother. 2025 Jun 4;74(7):234. doi: 10.1007/s00262-025-04072-6 (PMC12137825; doi:10.1007/s00262-025-04072-6)
Supplement: Supplementary file 2 — Supplementary file2 (PDF 114 kb) [file 262_2025_4072_MOESM2_ESM.pdf]

Target	Target species	Clone	Conjugation	Manufacturer
PD-L1	Human	29E.2A3	BV650	BD Biosciences
PD-L2	Human	MIH18	BV711	BD Biosciences
CD80	Human	2D10.4	BV510	BD Biosciences
CD86	Human	2331	BV605	BD Biosciences
B7-H2	Human	2D3/B7-H2	BV421	BD Biosciences
B7-H3	Human	MIH42	PE-Cy7	Biolegend
B7-H4	Human	MIH43	PE	BD Biosciences
B7-H5	Human	MIH65.rMAb	PE-CF594	BD Biosciences
B7-H6	Human	JAM1EW	PerCP-eFluor 710	Thermo Fisher Scientific
CD3	Human	SK7	AF700	BD Biosciences
CD4	Human	RPA-T4	APC-Cy7	BD Biosciences
CD8	Human	RPA-T8	FITC	BD Biosciences
PD-1	Human	EH12.1	BB700	BD Biosciences
EOMES	Human	X4-83	PE-CF594	BD Biosciences
TBET	Human	4B10	PE	BD Biosciences
FOXP3	Human	236A/E7	APC	Thermo Fisher Scientific
CD4	Mouse	GK1.5	PE-Cy7	Thermo Fisher Scientific
CD8	Rat	341	PE	Thermo Fisher Scientific
MHC-II	Mouse/rat	14-4-4S	FITC	Thermo Fisher Scientific

Supplementary Table 1. Antibodies used in the study.
