# Supplementary material for: Oncolytic adenovirus encoding variant interleukin-2 combined with chemotherapy enables PD-L1 inhibition in pancreatic cancer models
Source: Cancer Immunol Immunother. 2025 Jun 4;74(7):234. doi: 10.1007/s00262-025-04072-6 (PMC12137825; doi:10.1007/s00262-025-04072-6)
Supplement: Supplementary file 3 — Supplementary file3 (PDF 71 kb) [file 262_2025_4072_MOESM3_ESM.pdf]

Primer/probe	Sequence
Hexon_FW	CCTACACCAACACAACAACTC
Hexon_RV	ATCCACCTCAAAAGTCATGTC
Hexon_Probe	[FAM]AAACCTTCTCTACGCCAACTCCGCCCA[TAM]
Beta-actin_FW	TCACCCACACTGTGCCCATCT
Beta-actin_RV	GTGAGGATCTTCATGAGGTAGTCAGTC
Beta-actin_Probe	[FAM]ATGCCCTCCCCCATGCCATCCTGCGT[TAM]

Supplementary Table 2. Primers and probes used in the study.
